# Primary Auxin Response Genes *GH3s* and *DAO1* Modulate Stamen Elongation in *Arabidopsis thaliana* and *Solanum lycopersicum*

**DOI:** 10.1111/ppl.70340

**Published:** 2025-06-18

**Authors:** Davide Marzi, Maria Luisa Antenozio, Roberta Ghelli, Valentina Cecchetti, Francesca Romana Iacobini, Marzia Beccaccioli, Massimo Reverberi, Maurizio Enea Picarella, Andrea Mazzucato, Patrizia Brunetti, Maura Cardarelli

**Affiliations:** ^1^ Research Institute on Terrestrial Ecosystems‐National Research Council (IRET‐CNR) Rome Italy; ^2^ National Biodiversity Future Center (NBFC) Palermo Italy; ^3^ Institute of Molecular Biology and Pathology‐National Research Council (IBPM‐CNR) Sapienza University of Rome Rome Italy; ^4^ Department of Environmental Biology (DBA) Sapienza University of Rome Rome Italy; ^5^ Department of Biology University of Fribourg Fribourg Switzerland; ^6^ Department of Agriculture and Forest Sciences (DAFNE) Tuscia University Viterbo Italy

**Keywords:** auxin, flower development, gene expression, jasmonic acid, male fertility, stamen

## Abstract

Late stamen development is mainly regulated by auxin, which acts through the Auxin Response Factor 8 (ARF8) to promote stamen elongation in *Arabidopsis thaliana* . Auxin concentration in the stamens peaks at the beginning of late development and then declines, but the contribution of auxin inactivation to auxin homeostasis in stamen has never been determined. In this study, we show that the expression of the acyl amido synthetases *AtGH3.3*, *AtGH3.5*, and *AtGH3.6* (*AtGH3s*), which are involved in the conjugation of indole‐3‐acetic acid (IAA) with amino acids, is repressed, while the IAA oxidase *AtDAO1* is upregulated in Arabidopsis *arf8‐7* stamens. The *gh3s* triple mutants exhibit a 24% increased stamen length, while *gh3s* and *dao1* single mutants develop stamens with a reduced length of about 4% and 21%, respectively. We show that *AtGH3s* transcription is upregulated in *dao1* stamens, whereas *AtDAO1* expression is reduced in *gh3s* triple mutant stamens. Consistent with the long stamen phenotype, *gh3s* flower buds contain increased levels of free IAA and decreased levels of IAA‐Asp. We also show that genes orthologous to *AtARF8*, *AtGH3s* and *AtDAO1* are expressed in tomato stamens during flower development. We show that *SlGH3s* expression is mainly increased, while that of *SlARF8A*, *SlARF8B* and *SlDAO2* is decreased in stamens from the parthenocarpic fruit (*pat*) mutant, which develops stamens of reduced length. Accordingly, free IAA levels decrease while IAA‐Asp levels increase in tomato stamens. Collectively, our results demonstrate that *GH3s* and *DAO1* modulate auxin homeostasis and regulate stamen development in both Arabidopsis and tomato.

## 1 Introduction

In *Arabidopsis thaliana* , auxin controls and coordinates the three processes that occur in the stamens after male meiosis, during the late development phase: filament elongation, pollen maturation, and anther dehiscence. Auxin promotes filament elongation while playing a negative role in pollen maturation and anther dehiscence (Cardarelli and Ghelli 2020; Cecchetti et al. 2008). This control is achieved by changes in auxin concentration and localization, based on biosynthesis and transport in the stamen tissues during subsequent developmental stages (Cardarelli and Cecchetti 2014; Liu, Ghelli, et al. 2022). Auxin synthesis occurs at pre‐meiotic and meiotic stages (stages 8 and 9) and at the beginning of late development (stage 10), leading to a peak at stage 10 in the middle layer inside anthers, which is required for the successive proper distribution of IAA to the tapetum, endothecium, and procambial cells (Cecchetti et al. 2017, 2008). The high concentration of auxin at this stage avoids premature pollen maturation and anther dehiscence while inducing the slow phase of filament elongation (Cecchetti et al. 2017, 2008; Ghelli et al. 2018; Liu, Ghelli, et al. 2022). As the tapetum begins to degenerate (late stage 11), auxin concentration decreases, and auxin is transported basipetally from the procambium toward the basal side of the stamen, inducing the rapid phase of filament elongation (stage 12; Ghelli et al. 2018; Tashiro et al. 2009). The decrease in auxin levels allows the beginning of pollen maturation and anther dehiscence by inducing the biosynthesis of jasmonic acid (JA), which is required for pollen maturation and anther opening (Cecchetti et al. 2013). In addition, JA also positively contributes to the rapid phase of filament elongation (stage 12) just before anthesis, when the stamens reach pistil length (stage 13). Stamen elongation is due to the expansion of the filament cells and continues after anthesis, when the filaments become longer than the pistil and reach their maximum length at stage 14 (Cecchetti et al. 2008; Marzi et al. 2020).

Auxin acts through Auxin Response Factors (ARFs), a family of transcription factors that regulate the expression of several hormone‐response genes. In Arabidopsis, *ARF8* and *ARF6* play a central role in controlling late stamen development: *arf6 arf8* double mutants have short stamen filaments, non‐viable pollen grains, indehiscent anthers, and low JA production in flower buds (Nagpal et al. 2005; Tabata et al. 2010). Recently, we have shown that ARF8 plays a key role in late stamen development through its splice variants ARF8.4 (and to a lesser extent ARF8.2), which mainly controls stamen elongation and anther dehiscence, whereas the full‐length form ARF8.1 is involved in pollen maturation and tapetum development (Ghelli et al. 2023, 2018).

In addition to auxin biosynthesis and transport, auxin inactivation through conjugation and oxidation of IAA is known to modulate auxin concentration in plants; however, little is known about their role during stamen development (Guo et al. 2022; Gutierrez et al. 2012; Porco et al. 2016). Among the ARFs regulated genes, a subset of group II auxin‐induced Gretchen Hagen 3 (GH3) family members has been identified as acyl acid amido synthetase. This enzyme conjugates various amino acids with auxin, leading to an increase in inactive forms of IAA. In particular, auxin conjugates formed mainly with aspartate and glutamate, IAA‐Asp and IAA‐Glu, are accumulated as reversible storage forms of IAA or can be degraded (Hayashi et al. 2021). This GH3‐degradation pathway is mainly observed after high levels of IAA during lateral root emergence, which induces the transcription of *GH3* genes (Mellor et al. 2016). During root growth in Arabidopsis, ARF8 controls auxin levels through the transcriptional regulation of the *GH3.6*, *GH3.5*, and *GH3.17* genes (Gutierrez et al. 2012). However, the role of *GH3* genes does not always seem to be related to IAA homeostasis, since *GH3.3*, *GH3.5*, and *GH3.6* also modulate JA homeostasis to control adventitious root formation (Gutierrez et al. 2012). IAA oxidation by Dioxygenase for Auxin Oxidation1 (DAO1) has long been considered an alternative auxin degradation pathway (Lakehal, Dob, et al. 2019; Mellor et al. 2016; Porco et al. 2016). However, Hayashi et al. (2021) demonstrated that AtDAO1 acts downstream of AtGH3 in the same pathway, as AtDAO1 irreversibly oxidizes IAA‐Asp and IAA‐Glu to oxIAA‐Asp and oxIAA‐Glu, respectively, thereby regulating the levels of the storage forms in Arabidopsis. Interestingly, *dao1* mutant lines have shorter siliques suggesting a role for this gene in reproductive phases, but no correlation between DAO1 and stamen development has been reported (Porco et al. 2016; Zhang and Peer 2017). However, *DAO1* transcription is dependent on JA levels, which are in turn regulated by ARF8‐mediated auxin signaling, suggesting a tight connection between ARF8 and DAO1 (Gutierrez et al. 2012; Lakehal, Chaabouni, et al. 2019; Lakehal, Dob, et al. 2019; Nagpal et al. 2005). Furthermore, several authors have shown that *DAO1* and *GH3* genes have a compensatory expression when at least one of these factors is missing, supporting the idea of a network based on *ARF8*, *GH3* genes, and *DAO1* (Campos 2021; Mellor et al. 2016; Porco et al. 2016; Zhang et al. 2016).

In contrast to Arabidopsis, auxin levels and auxin responses in stamens have been less studied in other species. In tomato (*Solanum licopersicum* L.), the role of auxin in stamen development and male fertility is not yet fully understood, with most of the available information being indirect and derived from experiments focused on fruit development (Goldental‐Cohen et al. 2017; Gorguet et al. 2005; Pattison and Catalá 2012). However, in tomato anthers at developmental stage 0, at the end of male meiosis (corresponding to stage 10 in Arabidopsis), auxin accumulation as assessed by DR5‐Venus analysis showed the appearance of a specific signal in the tapetum and inner tapetum (Goldental‐Cohen et al. 2017). In addition, *ARF* genes also appear to be involved in stamen development, as the downregulation of the putative ARF6 and ARF8 orthologs, through the action of the Arabidopsis mir167, leads to floral developmental defects, including reduced stamen length and the absence of pollen germination on the stigma (Goetz et al. 2007; Liu et al. 2014). Furthermore, two different lines of evidence suggest that auxin concentration is related to male fertility in tomato plants: (i) different PIN family members involved in auxin transport are expressed during flower development, as suggested by gene expression analysis of tomato flower organs (Pattison and Catalá 2012) and (ii), the photoperiod‐sensitive male‐sterile mutant 7B‐1 has lower endogenous IAA levels and higher expression of *SlARF8* (Omidvar and Fellner 2015). In addition, the parthenocarpic fruit (*pat*) mutant is male‐sterile and has defective stamens with reduced stamen length and altered IAA levels in the ovary (Gorguet et al. 2005; Johkan et al. 2010; Mazzucato et al. 1998). The *pat* mutation has been associated with genetic lesions at the class III homeodomain leucine‐zipper transcription factor SlHB15A, that represses auxin signaling in tomato flower development (Clepet et al. 2021; Picarella et al. 2024). Recent evidence also suggests a role for JA in stamen development, as tomato mutants defective in JA perception show altered male fertility (Dobritzsch et al. 2015). For the *GH3* genes, 15 different members of the family have been identified in tomato, and group II SlGH3 paralogs show an auxin‐responsive expression profile in roots (Liao et al. 2015). Interestingly, a role for SlARF8A and SlARF8B in regulating conjugated auxin levels, including IAA‐Asp, through the regulation of SlGH3.4, has been demonstrated in tomato fruits during the development of locular and placental tissues (Hua et al. 2024).

Understanding the role of auxin in stamen development is crucial for a species of agronomic interest such as tomato, where male sterility is used for large‐scale production of hybrid seed.

In this study, to determine the role of the main auxin inactivation pathway in late stamen development, we found by qRT‐PCR analysis that the expression of *AtGH3.3*, *AtGH3.5*, *AtGH3.6* is downregulated in *arf8‐7* stamens, while that of *AtDAO1* is upregulated. By functional and expression analysis of *AtGH3s* and *AtDAO1*, and by measurement of auxin and JA levels, we demonstrated that in Arabidopsis these genes modulate only auxin levels in the stamens through a feedback loop that affects their respective expression. By analyzing the expression of genes homologous to *AtARF8, AtGH3.3, AtGH3.5, AtGH3.6*, and *AtDAO1* in tomato wild type (Chico III) and *pat* mutant stamens and by measuring auxin and JA levels, we showed that they are expressed during stamen development. We found a deregulation of *SlGH3s* and a down‐regulation of *SlDAO1* expression in *pat* mutant stamens that correlates with reduced auxin content, increased IAA‐Asp concentration, and elevated JA levels.

## 2 Materials and Methods

### 2.1 Plant Materials and Growth Conditions

All the *Arabidopsis thaliana* L. lines used in this research are in the Col‐0 background. Arabidopsis mutant lines *arf8*‐7 have been previously described in (Ghelli et al. 2018; Gutierrez et al. 2012). Arabidopsis *gh3.3‐1* (*SM_3‐39271*), *gh3.5‐2* (*SALK_151766*), *gh3.6‐1* (*SALK_133707*), single knockout mutant, *gh3.3‐1gh3.5‐2*, *gh3.3‐1gh3.6‐1*, and *gh3.5‐2gh3.6‐1* double mutants, and *gh3.3‐1gh3.5‐2gh3.6‐1* triple mutants have been characterized and described in (Gutierrez et al. 2012). Arabidopsis mutant lines *dao1‐1* (Salk_093162) and *dao1‐3* (Salk_082522) were obtained from the Arabidopsis Biological Resource Center (ABRC). Homozygous mutant lines were identified by PCR using the primers listed in Table S1.

Arabidopsis seeds were surface sterilized and stratified in water in the dark for 3 days at 4°C, then sown and grown on solid ½ Murashige and Skoog medium, supplemented with 1% sucrose and 0.8% agar, for 7 days in a 16 h‐light/8 h‐dark cycle at 24°C/21°C, with a light intensity of 130 μmol m^−2^ s^−1^. The seedlings were then transferred to soil pots containing universal potting soil and plants were grown in the same growth chamber until flowering for 4 weeks.

*Solanum lycopersicum* L. mutant parthenocarpic fruit (*pat*) lines have been previously characterized (Beraldi et al. 2004; Mazzucato et al. 1998; Picarella et al. 2024; Ruiu et al. 2015) and are in the Chico III cv. background.

Tomato Chico III and *pat* seeds were sown in Petri dishes with moistened paper. After germination, the plantlets were transferred to 7 cm plastic pots containing loam‐sand‐peat (1:1:2). At the fifth leaf stage, the plantlets were transplanted into 25 cm plastic pots and fertilized with a commercial fertilizer (N‐P‐K 21‐7‐14, plus oligoelements) and transferred to greenhouse conditions, under a 16 h‐light/8 h‐dark cycle at 26° ± 1°/18°C ± 1°C.

### 2.2 Phenotypical Analyses

Flower developmental stages in Arabidopsis were determined as previously described (Cecchetti et al. 2008; Marzi et al. 2020), while flower developmental stages in tomato were determined according to the literature (Mazzucato et al. 1998).

Sepals and petals were removed from flowers and stamen length was determined by measuring from the base of the stamen filament to the tip of the anther at stage 14 of flower development in Arabidopsis, while it was measured at the beginning of stage 3 (flower disclosure) in tomato. About 10–30 flowers obtained from six independent biological replicates per line were analyzed. Images were captured using a stereomicroscope equipped with a ProgRes C3 digital camera and stamen length was measured using ImageJ (https://imagej.nih.gov/ij/).

### 2.3 Identification of Arabidopsis Orthologous Genes in *Solanum lycopersicum*

The members of the tomato *AtGH3* gene family were identified by using AtGH3.3, AtGH3.5, and AtGH3.6 protein sequences as queries for blastp with Sol genomics and JGI Phytozome Networks (https://solgenomics.net/tools/blast/; https://phytozome‐next.jgi.doe.gov). The analysis was performed using the database Tomato genome protein sequences (ITAG release 2.40), and the following basic parameters: e‐value 1e‐10, score > 900, substitution matrix BLOSUM62 (default) and IDentity % > 70%. As described in Tables S2 and S3, SlGH3.2, SlGH3.3, and SlGH3.4 proteins showed the higher identity to AtGH3.3 (76.63%, 73.91%, 73.38%, respectively), while the SlGH3.9, SlGH3.15, and SlGH3.7 proteins showed the highest identity to AtGH3.5 and AtGH3.6 (SlGH3.9 with 81.7% and 83.01% of identity to AtGH3.5 and AtGH3.6, and SlGH3.15 with 79.12% and 80.42% to AtGH3.5 and AtGH3.6, SlGH3.7 with 72.79% and 74.41% to ATGH3,5 and AtGH3.6, respectively). SlGH3.7 was excluded from subsequent analysis because this gene responds relatively slowly to IAA, compared to the other SlGH3s and AtGH3s gene members that respond rapidly to auxin (Liao et al. 2015).

SlDAO2 (Solyc02g068320.2) was selected for its higher homology to both AtDAO1 and AtDAO2 (Table S3), compared to SlDAO1. In addition, *SlDAO2* expression has been detected in flower buds and during hypocotyl elongation (Lei et al. 2023).

Both SlARF8A (Solyc03g031970.2) and SlARF8B (Solyc02g037530.2) proteins were selected based on their identity to the protein sequence of AtARF8 (Table S3).

### 2.4 RNA Extraction and Gene Expression

Gene expression was evaluated by means of qRT‐PCR, as previously described (Antenozio et al. 2022; Marzi et al. 2020). Total RNA was obtained from stamens collected from about 50 flowers per stage, for each genotype. In Arabidopsis, each flower contains 5–6 stamens, of which four are long stamens and the remaining 1–2 are short stamens. Only the four long stamens were collected for qRT‐PCR analysis, resulting in about 200 stamens collected for RNA extraction for each genotype. Total RNA was extracted using the RNeasy Plant Mini Kit (Qiagen) and reverse‐transcribed using a QuantiTect Reverse Transcription Kit (Qiagen). SYBR Green‐based quantitative assays were performed by using a Rotor‐Gene Q and analyzed using the Rotor‐Gene Q 2.3.1 software. cDNAs were amplified using the primers listed in Table S1. Gene expression levels were normalized to the levels of *ACTIN8* (*ACT8*) for Arabidopsis and Clathrin adaptor complex subunit protein (*CAC*, *Solyc08g006960*) for tomato. Data analysis was performed using the 2^−ΔCt^ or 2^−ΔΔCt^ method according to literature (Livak and Schmittgen 2001; Pfaffl 2001). All quantifications were performed in three biological replicates, each consisting of technical triplicates.

### 2.5 Quantification of IAA, JA, and IAA‐Asp Concentration

Chemical analyses were performed on 20 mg of flower buds or stamens, depending on plant species. For Arabidopsis, 20 mg of flower buds from stage 10–11 were pooled for each genotype, while for tomato, 20 mg of stamens from stages 1–2 were pooled per genotype. Plant material was rapidly frozen in liquid nitrogen. Up to three replicates from three independent experiments were harvested. Hormone extraction and the analysis method are reported in Beccaccioli et al. (2021) with some adjustments.

Flower buds or stamens have been powdered in the presence of liquid nitrogen, to each sample, 750 μL of extraction buffer consisted of: methanol: water: acetic acid (90:9:1, v/v/v) were added. Afterwards, 5 μL of the internal standard 1‐naphthaleneacetic acid (NAA, 100 μM) was added, and then the samples were vortexed for 5 min and centrifuged for 10 min at 4°C at 5590 g. The upper phase was kept, and a second extraction was repeated on buds/stamen by adding another 750 μL of extraction buffer; then the samples were vortexed and centrifuged as described above. The pooled extracts were dried under a nitrogen stream and dissolved in 100 μL of water containing 0.05% acetic acid. From that, 10 μL of each extract was analysed by mass spectrometry.

Mass spectrometry analyses were performed by LC (HPLC 1200 series rapid resolution) coupled to a triple quadrupole MS (G6420 series triple quadrupole, QqQ; Agilent Technologies), with an electrospray ionization source (ESI). The chromatographic column (Zorbax ECLIPSE XDB‐C18 rapid resolution HT 4.6 × 50 mm 1.8 μm p.s.) was set to 25°C, mobile phases consisted of phase A (water containing 0.05% acetic) and B (acetonitrile). The elution gradient was as follows: 0–3 min 15% B, 3–5 min 100% B, 5–6 min 100% B, 6–7 min 15% B, and 7–8 min 15% B. The gradient was followed by 5 min of the original mobile phase for re‐equilibration with a constant flow rate of 0.6 mL min^−1^. For the QqQ the temperature was set to 350°C, nitrogen flow to 8 L min^−1^, nebulization pressure was at 20 psi, and voltage at 4000 V. Multiple reaction monitoring (MRM) conditions for the LC‐ESI‐MS/MS analysis of IAA, JA and IAA‐Asp are reported in the Table S4. Analysis software was provided by Agilent Technologies.

The analyses were performed as reported previously by comparing the peak area of the plant hormones normalized by the peak area of the internal standard (NAA) for each hormone extracted, providing a relative value amount (Badiali et al. 2024; Beccaccioli et al. 2021).

### 2.6 Statistical Analysis

Statistical analyses were performed using GraphPad Prism 8. Differences between two groups were assessed using the Student's *t*‐test and asterisks indicate the significant differences (*p < 0.05, **p < 0.01, ***p < 0.001). One‐way or two‐way analysis of variance (ANOVA) followed by Tukey's multiple comparison tests were used depending on the number of variables considered at the same time, such as stamen length, flower developmental stage, genotype and gene expression. Different letters indicate significant differences between groups (*p* < 0.05).

## 3 Results

### 3.1 *GH3.3*, *GH3.5*, and *GH3.6* are Expressed in Stamens and Downregulated in *arf8‐7* Mutants in Arabidopsis

We have previously shown that stamens from the Auxin Response Factor 8 (ARF8) mutant line *arf8‐7* have reduced length and reduced expression of *AtGH3.3*, which is known to have redundant functions with *GH3.5* and *GH3.6* in roots (Ghelli et al. 2018; Gutierrez et al. 2012). Thus, we analyzed the expression of *GH3.3*, *GH3.5* and *GH3.6* (hereafter referred to as *GH3s*) in stamens of wild type (Col‐0) and *arf8‐7* mutants at stages 10–11 pooled together, when auxin concentration is high (Cecchetti et al. 2008). We confirmed the downregulation of *AtGH3.3* and found that the expression of *AtGH3.5* and *AtGH3.6* was also significantly lower, about 2.5, 2.4 and 4.7 fold respectively, compared to Col‐0 (Figure S1).

**FIGURE 1:**
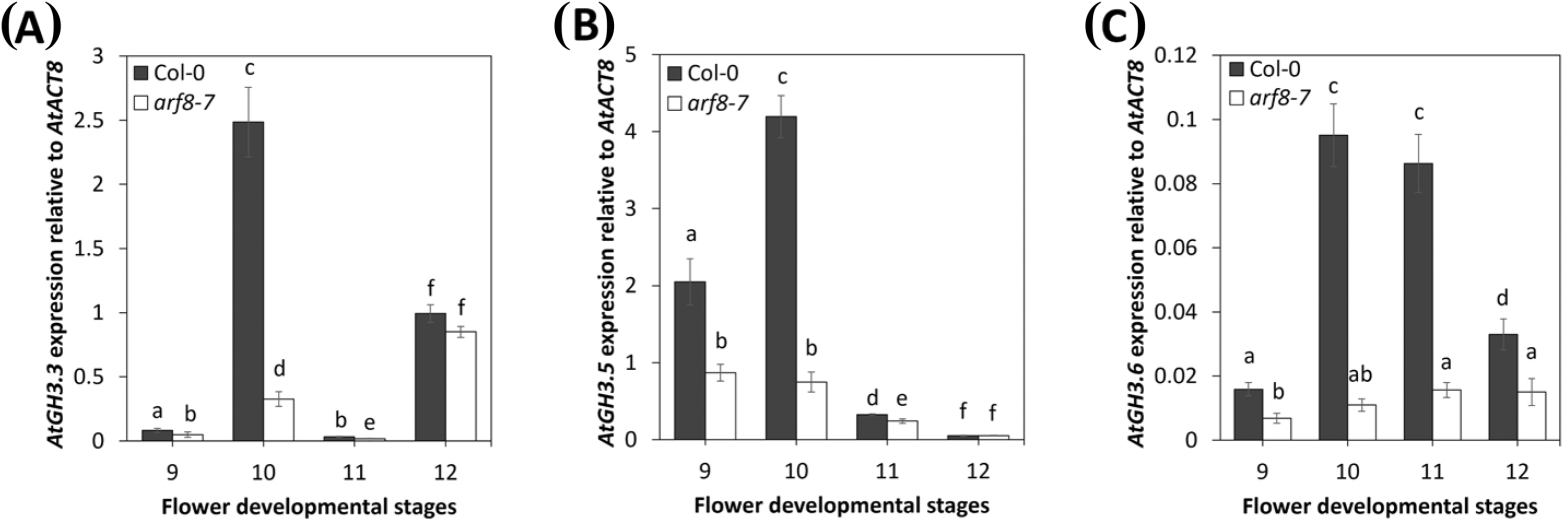
*AtGH3s* expression in Arabidopsis wild type (Col‐0) and *arf8‐7* stamens. qRT‐PCR analysis of *AtGH3.3* (A), *AtGH3.5* (B) and *AtGH3.6* (C) expression in wild type (Col‐0) and *arf8‐7* mutant stamens at stages from 9 to 12 of flower development. Genes expression is presented relative to the reference gene *AtACT8*. The values shown are means ± SD (*n* = 3). Bar colors indicate different lines (i.e., Col‐0, black; *arf8‐7*, white). Different letters represent significant differences as determined by two‐way ANOVA followed by Tukey's test (*p* < 0.05).

To gain insights into the expression profile of *AtGH3s*, we analyzed *AtGH3.3*, *AtGH3.5* and *AtGH3.6* expression from stage 9 to stage 12 of flower development in Col‐0 and *arf8‐7* stamens. As shown in Figure 1A, in Col‐0 stamens *AtGH3.3* expression was low at stage 9, peaked at stage 10, decreased at stage 11, and increased again at stage 12. *AtGH3.3* expression in *arf8‐7* stamens showed a similar trend to that of Col‐0. However, it was significantly lower at stages 9, 10 and 11 of flower development compared to Col‐0 (Figure 1A). *AtGH3.5* expression in Col‐0 stamens increased from stage 9 to stage 10 and significantly decreased at stages 11 and 12 (Figure 1B). In *arf8‐7* stamens, *AtGH3.5* expression was lower than Col‐0 at stages 9, 10 and 11 of flower development (Figure 1B). In Col‐0 stamens *AtGH3.6* expression increased from stage 9 to 10, remained high at stage 11 and then decreased at stage 12 (Figure 1C). Compared to Col‐0, in *arf8‐7* stamens *AtGH3.6* expression was significantly lower from stage 9 to 12 (Figure 1C).

These results show that *AtGH3s* are actively transcribed in stamens, with partially overlapping expression profiles, and are all downregulated in *arf8‐7* stamens mainly at early stages of late flower development.

### 3.2 Mutations in *GH3s* Affect Stamen Elongation and *GH3s* Gene Expression in Arabidopsis

**FIGURE 2:**
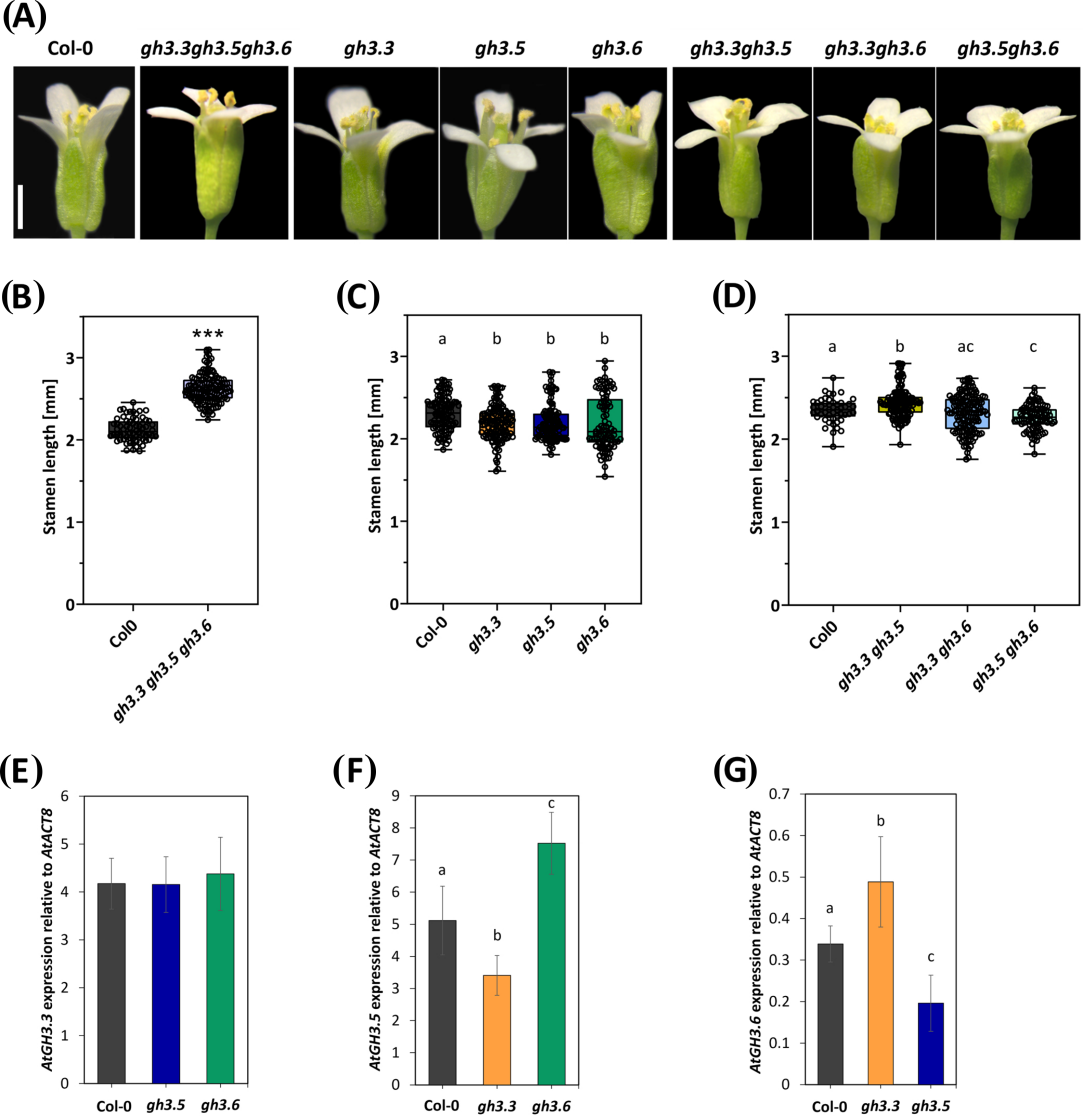
Stamen length analysis and *AtGH3s* expression in *gh3s* and wild type (Col‐0). Rapresentative images of flower buds at stage 14 of flower development in wild type (Col‐0) and *gh3s* triple, single and double mutant lines (A). Box plot with dots of stamen length measured at stage 14 of flower development in wild type (Col‐0) and *gh3s* triple (A), single (B) and double (C) mutant lines. In (A) statistically significant differences were determined on raw data by Student's *T* test and indicated by asterisks (***p < 0.001). In (B–D), different letters represent significant differences as determined by one‐way ANOVA followed by Tukey's test (*p* < 0.05). Scale bar = 1 mm. qRT‐PCR analysis of *AtGH3.3* (E), *AtGH3.5* (F) and *AtGH3.6* (G) expression in *gh3.3*, *gh3.5*, and *gh3.6* mutant and Col‐0 stamens at stages 10–11 (pooled together) of flower development. Genes expression is presented relative to the reference gene *AtACT8*. The values shown are means ± SD (*n* = 3). Bar colors indicate different lines (i.e., Col‐0, black; *gh3.3 gh3.5 gh3.6*, light violet; *gh3.3*, orange; *gh3.5*, blue; *gh3.6*, green; *gh3.3 gh3.5*, dark yellow; *gh3.3 gh3.6*, azure; *gh3.5 gh3.6*, light green). Different letters represent significant differences as determined by one‐way ANOVA followed by Tukey's test (*p* < 0.05).

To assess the contribution of *AtGH3s* to stamen development, we analyzed stamen length in *gh3.3 gh3.5 gh3.6* triple mutants at stage 14 of flower development, when stamens reach their maximum length (Marzi et al. 2020). As shown in Figure 2A,B, *gh3.3 gh3.5 gh3.6* stamens significantly increased in length by 24.8% compared to Col‐0. Furthermore, we measured stamen length in *gh3.3*, *gh3.5* and *gh3.6* single mutants, which showed a slight but significant reduction (5.2%, 4.0%, and 4.4%, respectively) compared to Col‐0 (Figure 2A,C). Due to the conflicting phenotypes between triple and single mutants, we also analyzed *gh3.3 gh3.5*, *gh3.3 gh3.6*, and *gh3.5 gh3.6* double mutants, which showed significantly longer stamens (3.9%), no differences and a significant reduction in stamen length (3.7%), respectively (Figure 2A,D).

The opposite stamen phenotype exhibited by single and double mutants compared to *gh3s* triple mutants could be due to compensatory regulation of *AtGH3.3*, *AtGH3.5*, or *AtGH3.6* expression by mutations in *AtGH3s*, as demonstrated for root development (Gutierrez et al. 2012). To verify this hypothesis, *AtGH3s* transcription was analyzed in *gh3.3*, *gh3.5*, and *gh3.6* single mutant stamens at stages 10–11 (pooled together), where *AtGH3s* are predominantly expressed. As shown in Figure 2E, *AtGH3.3* expression was unchanged in *gh3.5* and *gh3.6*. *AtGH3.5* transcript levels decreased in *gh3.3* while significantly increased in *gh3.6* (Figure 2F). *AtGH3.6* expression significantly increased in *gh3.3* while decreased in *gh3.5* (Figure 2G). Taken together, these results indicate that *AtGH3s* modulate stamen elongation and that mutations in *AtGH3s* genes activate a compensatory regulation of the expression of other *AtGH3s* in stamens.

### 3.3 *AtDAO1* Promotes Stamen Elongation and Its Expression Is Closely Linked to That of *AtGH3s* in Arabidopsis

**FIGURE 3:**
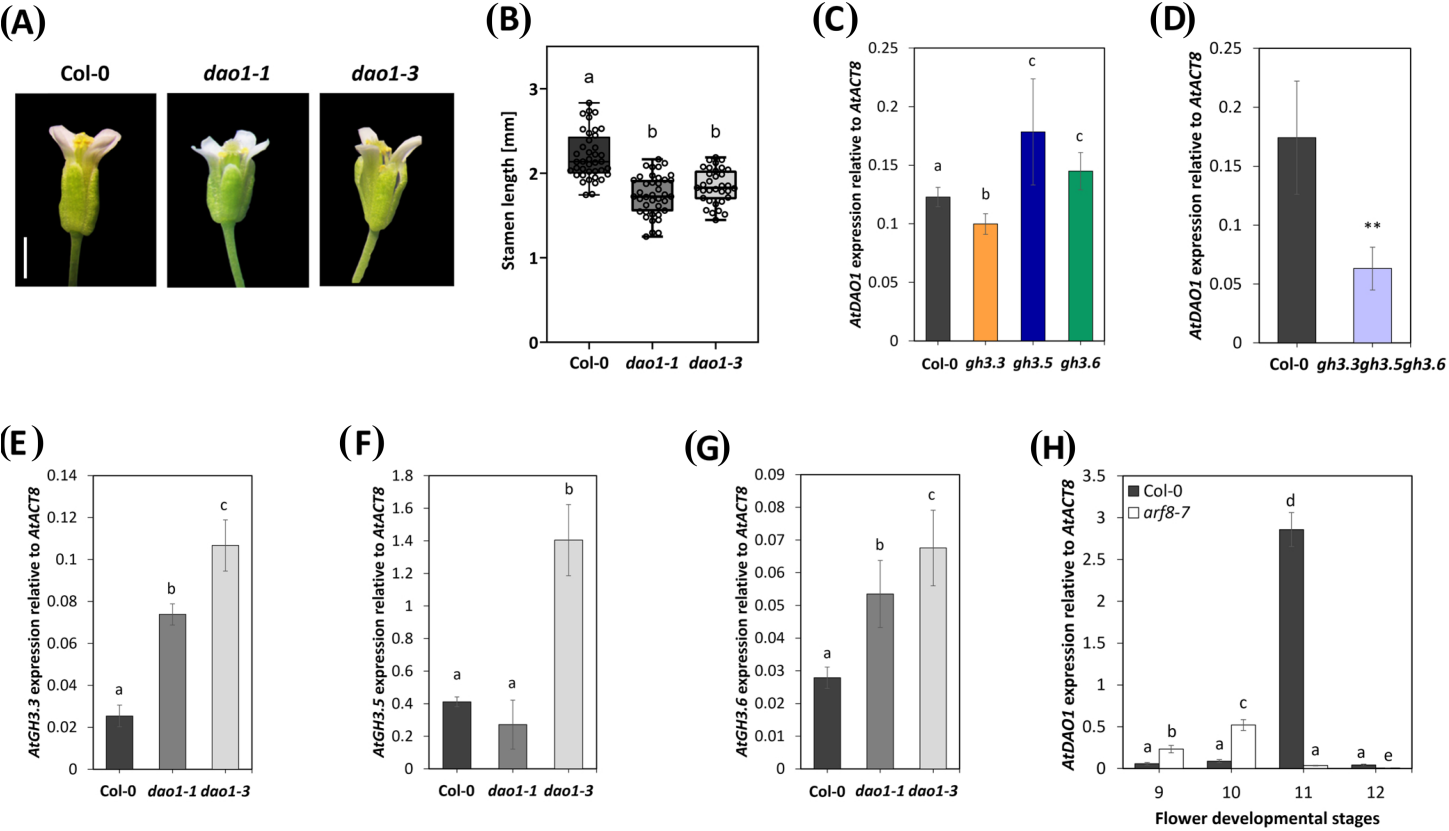
*AtGH3s* and *AtDAO1* expression in *gh3s*, *arf8‐7*, *dao1* and wild type (Col‐0) stamens. Rapresentative images of flower buds at stage 14 of flower development in wild type (Col‐0), and *dao1‐1* and *dao1‐3* mutants (A). Box plot with dots of stamen length measured at stage 14 of flower development in wild type (Col‐0), *dao1‐1* and *dao1‐3* mutant lines (B). Different letters represent significant differences as determined by one‐way ANOVA followed by Tukey's test (*p* < 0.05). Scale bar = 1 mm. qRT‐PCR analysis of *AtDAO1* expression in *gh3*.3, *gh3*.5, *gh3.6* single mutants (C), triple mutants (D) and Col‐0 stamens at stages 10–11 of flower development (pooled together). Analysis of *AtGH3.3* (E), *AtGH3.5* (F) and *AtGH3.6* (G) expression in *dao1‐1* and *dao1‐3* mutants and Col‐0 stamens at stages 10–11 (pooled together). Expression of *AtDAO1* in Col‐0 and *arf8‐7* mutant stamens at stages from 9 to 12 of flower development (H). Genes expression is presented relative to the reference gene *AtACT8*. The values shown are means ± SD (*n* = 3). Bar colors indicate different lines (i.e., Col‐0, black; *dao1‐1*, dark grey; *dao1‐3*, light gray; *gh3.3*, orange; *gh3.5*, blue; *gh3.6*, green; *gh3.3 gh3.5 gh3.6*, light violet; *arf8‐7*, white). Statistically significant differences were determined by Student's *T* test and indicated by asterisks (**p < 0.01) in (D). Different letters represent significant differences as determined by one‐way ANOVA followed by Tukey's test (*p* < 0.05) in (B and C) and (E–G), and by two‐way ANOVA followed by Tukey's test (*p* < 0.05) in (H).

The results described in the previous section do not explain the opposite stamen phenotype shown by single, double and triple mutants, suggesting that other players are involved in this process. This prompted us to analyze stamens of the mutant lines for *AtDAO1*, which encodes for an IAA‐oxidizing enzyme and is expressed in stamens (Zhang et al. 2016). In addition, the expression of *AtGH3.3* is upregulated in roots of *dao1* mutant lines (Mellor et al. 2016). We found a significant decrease in stamen length in both *dao1‐1* and *dao1‐3* mutant lines compared to Col‐0, by 21% and 16.2%, respectively (Figure 3A,B), indicating that *AtDAO1* promotes stamen elongation.

To assess whether there is an interplay between *AtDAO1* and *AtGH3s*, we analyzed *AtDAO1* transcript levels in *gh3.3, gh3.5*, and *gh3.6* single and triple mutant stamens at stages 10–11 (pooled together). As shown in Figure 3C, *AtDAO1* transcript was slightly but significantly decreased in *gh3.3*, whereas it was increased in *gh3.5* and *gh3.6* single mutant stamens. Similar to *gh3.3*, *AtDAO1* expression was significantly downregulated in *gh3.3*, *gh3.5*, *gh3.6* triple mutant stamens (Figure 3D). In addition, we analyzed the expression of *AtGH3s* in *dao1* mutant stamens. Both *AtGH3.3* and *AtGH3.6* were significantly upregulated in *dao1‐1* and *dao1‐3*, while *AtGH3.5* was unchanged in *dao1‐1* and significantly upregulated in *dao1‐3* (Figure 3E–G). Both *dao1‐1* and *dao1‐3* mutant lines contain a T‐DNA insertion located in the first exon and in the promoter, respectively. *AtDAO1* transcripts are not detected in *dao1‐1* and are reduced to 10% of the wild type in *dao1‐3* (Zhang et al. 2016), which could account for the differences observed in *AtGH3.5* expression.

To determine whether *AtDAO1* is expressed at the same stages as *AtGH3s* and whether it is regulated by ARF8, we analyzed *AtDAO1* transcript levels in Col‐0 and *arf8‐7* stamens at stages from 9 to 12 of flower development. As shown in Figure 3H, *AtDAO1* expression in Col‐0 is low at stages 9 and 10, increases at stage 11, when JA concentration peaks, and decreases at stage 12. This result is in contrast with *AtGH3.3* and *AtGH3.5* expression, which is mainly observed at stage 10, while is comparable to *AtGH3.6* expression, which increases at both stages 10 and 11 of flower development. Interestingly, in *arf8‐7* mutant stamens compared to Col‐0, *AtDAO1* expression significantly increases at stages 9 and 10, and decreases at stages 11 and 12 (Figure 3H), while that of *AtGH3s* is mainly downregulated from stage 9 to 12.

These results show that *AtDAO1* is predominantly expressed at later stages than *AtGH3s* and strongly suggest that both are regulated by *AtARF8*, while a compensatory expression of *AtGH3s* and *AtDAO1* occurs in *gh3s* and *dao1* mutants, likely modulating free IAA levels during stamen elongation.

### 3.4 Mutations in *GH3* Genes Affect Free IAA Levels in Arabidopsis Flower Buds

Both auxin and JA have a promotive effect on stamen elongation; therefore, *GH3s* could affect stamen development by interfering with endogenous levels of free IAA or JA, as in adventitious roots formation (Cardarelli and Cecchetti 2014; Gutierrez et al. 2012; Nagpal et al. 2005). To discern between these two possibilities, free IAA levels, IAA‐Asp content and JA levels, were evaluated in Col‐0 and *gh3.3 gh3.5 gh3.6* triple mutant flower buds at stages 10–12 (pooled together), in which IAA and JA content is detectable (Cecchetti et al. 2013; Nagpal et al. 2005).

**FIGURE 4:**
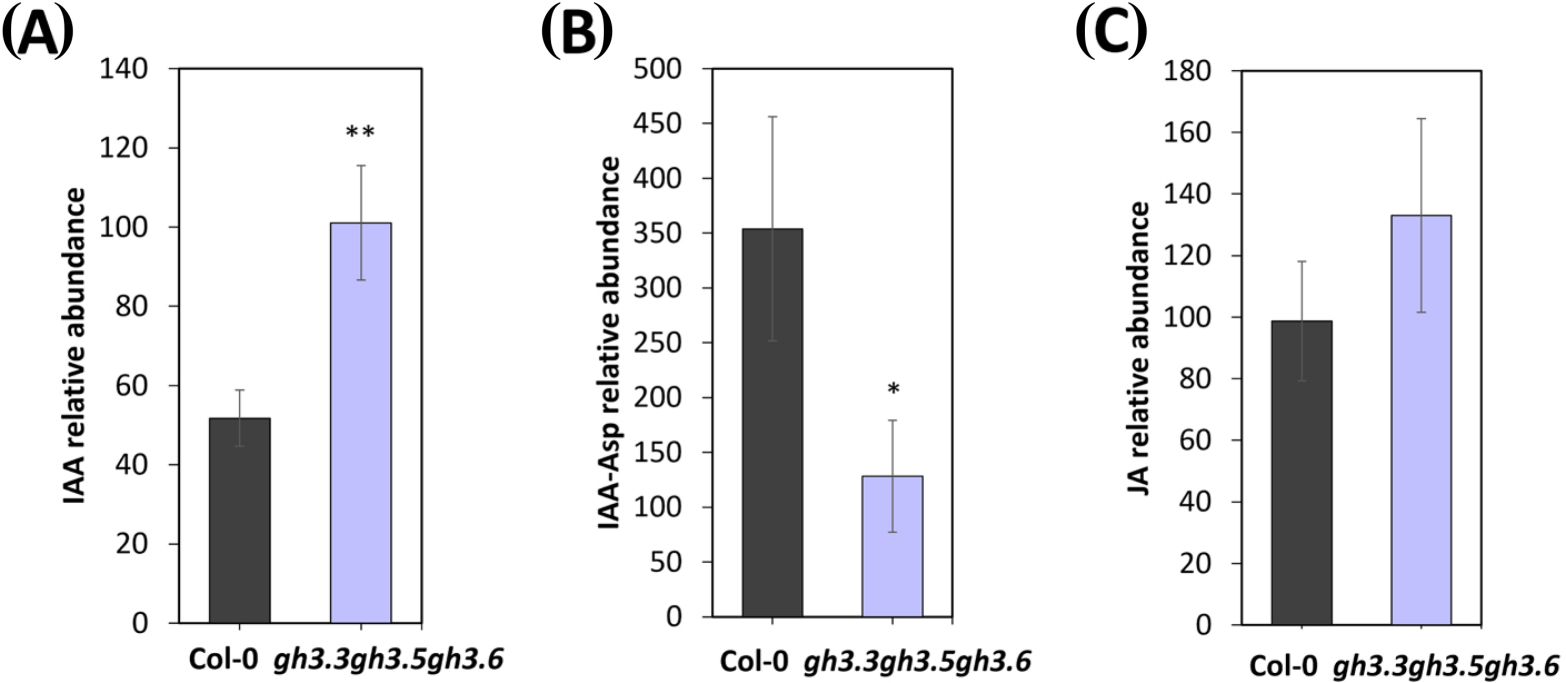
Analysis of IAA, IAA‐Asp and JA conce ntration in *gh3s* and wild type (Col‐0) flower buds. Hormones and hormones conjugate analysis was performed on 20 mg of flower buds at stages 10–11 pooled together of flower development, collected from *gh3s* triple mutant and wild type (Col‐0) lines. (A) Relative amount concentration of indole‐3‐acetic acid (IAA), (B) Indole‐3‐acetyl‐aspartate (IAA‐Asp) and (C) jasmonic acid (JA), was measured using HPLC coupled to a triple quadrupole MS equipped with an electrospray ionization source (ESI). Values are the means of three biological replicates ± SD. Bar colors indicate different lines (i.e., Col‐0, black; *gh3.3 gh3.5 gh3.6*, light violet). Statistically significant differences were determined by Student's *T* test and indicated by asterisks (*p < 0.05, **p < 0.01).

As shown in Figure 4A, IAA levels were significantly higher in *gh3.3*, *gh3.5*, and *gh3.6* triple mutant flower buds (about 2‐fold), while the IAA‐Asp amount showed a significant decrease (Figure 4B). In contrast, free JA levels slightly increased in the triple mutant flower buds, although no significant differences were observed compared to Col‐0 (Figure 4C).

These results indicate that the main function of *AtGH3s* in stamens is to conjugate free IAA and that their loss‐of‐function triggers the accumulation of free IAA, which could be responsible for the increased stamen length in the *gh3s* triple mutant lines.

### 3.5 Group II *SlGH3s* Genes and *SlDAO2* Are Expressed in Tomato Stamens During Development

Phylogenetic analysis showed that *AtGH3.3*, *AtGH3.5*, and *AtGH3.6* cluster in the same phylogenetic group II of *SlGH3.2*, *SlGH3.3*, *SlGH3.4*, *SlGH3.9*, and *SlGH3.15*. Among *SlGH3s*, *SlGH3.9* and *SlGH3.15* belong to the same subgroup as *AtGH3.5* and *AtGH3.6*, whereas *SlGH3.2*, *SlGH3.3*, and *SlGH3.4* belong to the same subgroup as *AtGH3.3* (Figure S2), as previously demonstrated (Kumar et al. 2012; Liao et al. 2015). By using AtGH3.3, AtGH3.5, and AtGH3.6 proteins as query sequences for blastp with Sol Genomics Network, we analyzed the degree of similarity between Arabidopsis and tomato sequences (Table S3). We found that all three SlGH3.2, SlGH3.4, and SlGH3.3 are highly homologous to AtGH3.3 (78.22%, 75.78%, and 75.67%, respectively), while SlGH3.9 and SlGH3.15 are highly homologous to both AtGH3.5 and AtGH3.6 (81.7% and 83.01%, respectively, and 81.35 and 79.87, respectively).

**FIGURE 5:**
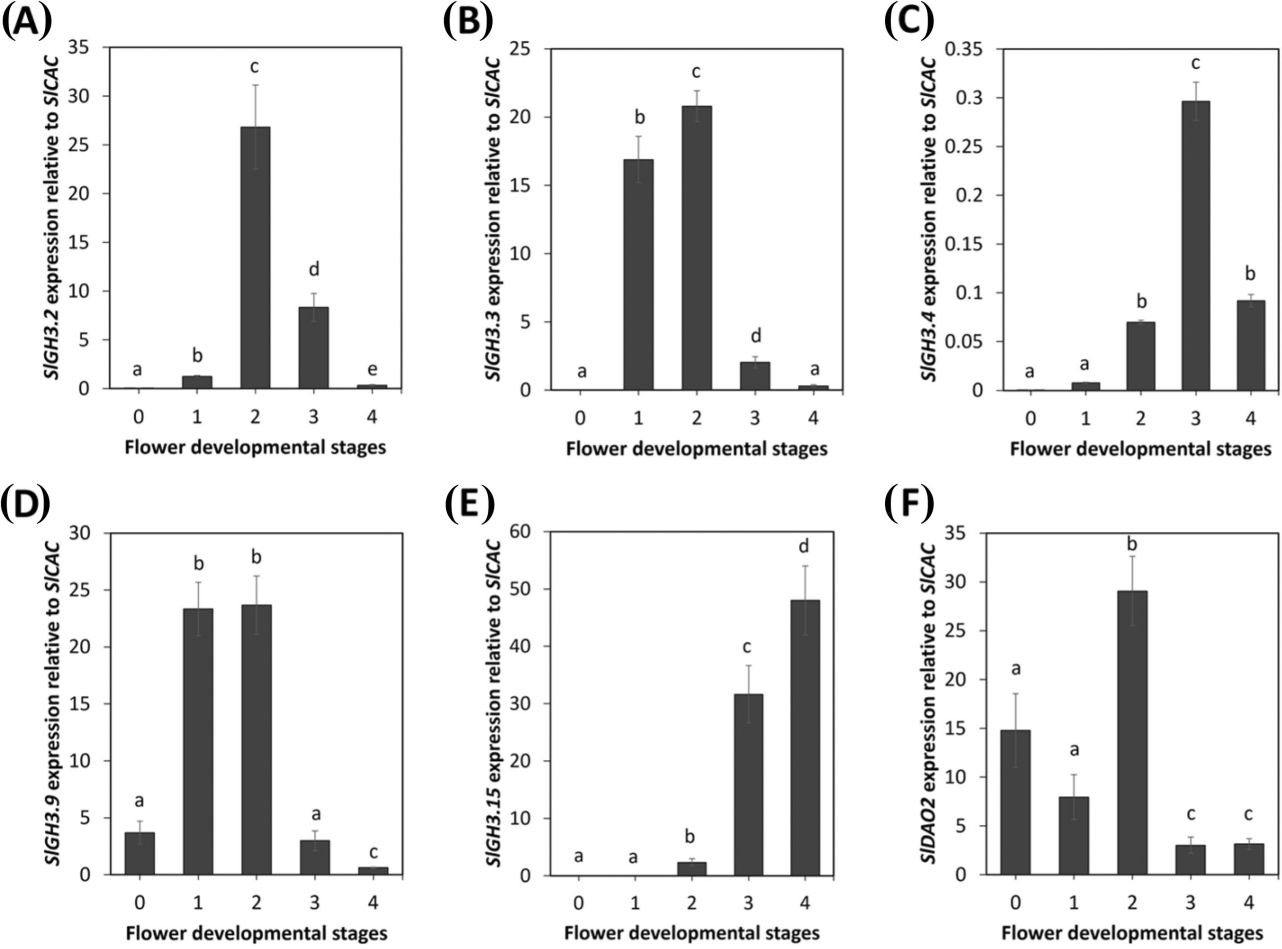
*SlGH3s* and *SlDAO2* expression in wild type tomato (Chico III) stamens. qRT‐PCR analysis of *SlGH3.2* (A), *SlGH3.3* (B), *SlGH3.4* (C), *SlGH3.9* (D), *SlGH3.15* (E) and *SlDAO2* (F) expression in tomato wild type (Chico III) stamens at stages from 0 to 4 of flower development. Genes expression is presented relative to the reference gene *SlCAC*. The values shown are means ± SD (*n* = 3). Different letters represent significant differences as determined by one‐way ANOVA followed by Tukey's test (*p* < 0.05).

To assess a possible involvement of these genes during stamen development in tomato, we analyzed their expression in stamens from stage 0 to stage 4, roughly corresponding to flower developmental stages 9–13 in Arabidopsis (Table S5; Brukhin et al. 2003; Cecchetti et al. 2008; Khaliluev et al. 2014; Marzi et al. 2020; Mazzucato et al. 1998; Smyth et al. 1990). As shown in Figure 5A, in wild type (Chico III) stamens, *SlGH3.2* was almost undetectable at stage 0, significantly increased from stage 1 to stage 2, while it decreased at stages 3 and 4. Similarly, the expression of *SlGH3.3* was almost undetectable at stage 0, increased at stages 1–2, and decreased at stages 3–4 (Figure 5B). The expression of *SlGH3.4* was almost undetectable at stages 0 and 1, increased from stage 2 to stage 3, and decreased at stage 4 (Figure 5C). The expression of *SlGH3.9* was low at stage 0, increased at stages 1–2, and decreased at stages 3 and 4 (Figure 5D), while that of *SlGH3.15* was barely detectable at stages 0–1 and significantly increased from stage 2 to stage 4 (Figure 5E).

Similarly, *SlDAO2* phylogenetic analysis showed a high homology to *AtDAO1* (59%; Table S3); thus, we evaluated its expression in stamens at stages from 0 to 4 of flower development. As shown in Figure 5F, in wild type stamens, *SlDAO2* expression peaks at stage 2 and significantly decreases at stages 3 and 4 of flower development.

These results show that all *SlGH3s* and *SlDAO2* are expressed during stamen development in tomato. *SlGH3.2*, *SlGH3.3* and *SlGH3.9* act predominantly at early stages (stages 1 and 2 corresponding to stages 10 and 11 in Arabidopsis), *SlGH3.4* and *SlGH3.15* at late stages (3 and 4 corresponding to stages 12 and 13 in Arabidopsis), while *SlDAO2* mainly is expressed at stage 0–2, corresponding to stage 9 and 11 in Arabidopsis.

### 3.6 *SlARF8A* and *SlARF8B* Expression Is Reduced in the Tomato Parthenocarpic *pat* Mutant

**FIGURE 6:**
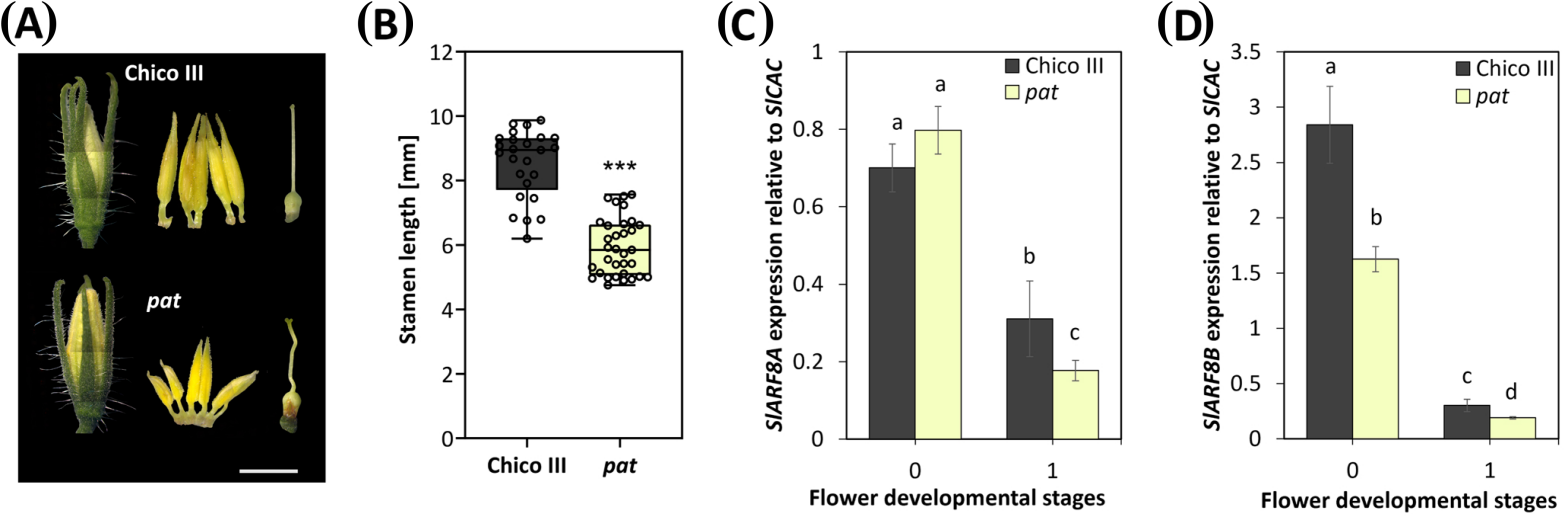
*pat* mutants show reduced stamen length and expression of *SlARF8A* and *SlARF8B*. Rapresentative images of flower buds at stage 3 of flower development in wild type (Chico III), and *pat* mutants (A). Stamen length in Chico III and *pat* (B). The values shown are means ± SD (*n* = 30) and statistically significant differences were determined by Student's *T* test and indicated by asterisks (***p < 0.001). Scale bar = 5 mm. qRT‐PCR analysis of *SlARF8A* (C) and *SlARF8B* (D) expression in *pat* mutants and Chico III stamens at stages 0 and 1 of flower development. Genes expression is presented relative to the reference gene *SlCAC*. The values shown are means ± SD (*n* = 3). Bar colors indicate different lines (i.e., Chico III, black; *pat*, light yellow). Different letters represent significant differences as determined by two‐way ANOVA followed by Tukey's test (*p* < 0.05).

The tomato parthenocarpic *pat* mutant shows an impaired stamen development, which affects plant fertility (Mazzucato et al. 1998). In agreement, we found that at the beginning of stage 3, when flower disclosure occurs, *pat* mutants developed significantly shorter stamens than the wild type (Chico III) (Figure 6A,B), similarly to *arf8‐7* mutants in Arabidopsis. Thus, we analyzed the expression of the two *AtARF8* homologs in tomato, namely *SlARF8A* and *SlARF8B* (Liu et al. 2014; Zouine et al. 2014), which show a high homology to the Arabidopsis gene (67% each, Table S3). As shown in Figure 6C,D, we found that in *pat* mutant stamens compared to wild type, *SlARF8A* transcript level is unchanged at stage 0 and significantly lower at stage 1, while *SlARF8B* expression is downregulated at both stages compared to Chico III (Figure 6C,D).

These results suggest that, similarly to Arabidopsis, *ARF8* plays a pivotal role during stamen development in tomato and that its downregulation results in decreased stamen length.

### 3.7 *SlGH3s* Genes and *SlDAO1* Expression Is Deregulated in the Tomato Parthenocarpic *pat* Mutant

**FIGURE 7:**
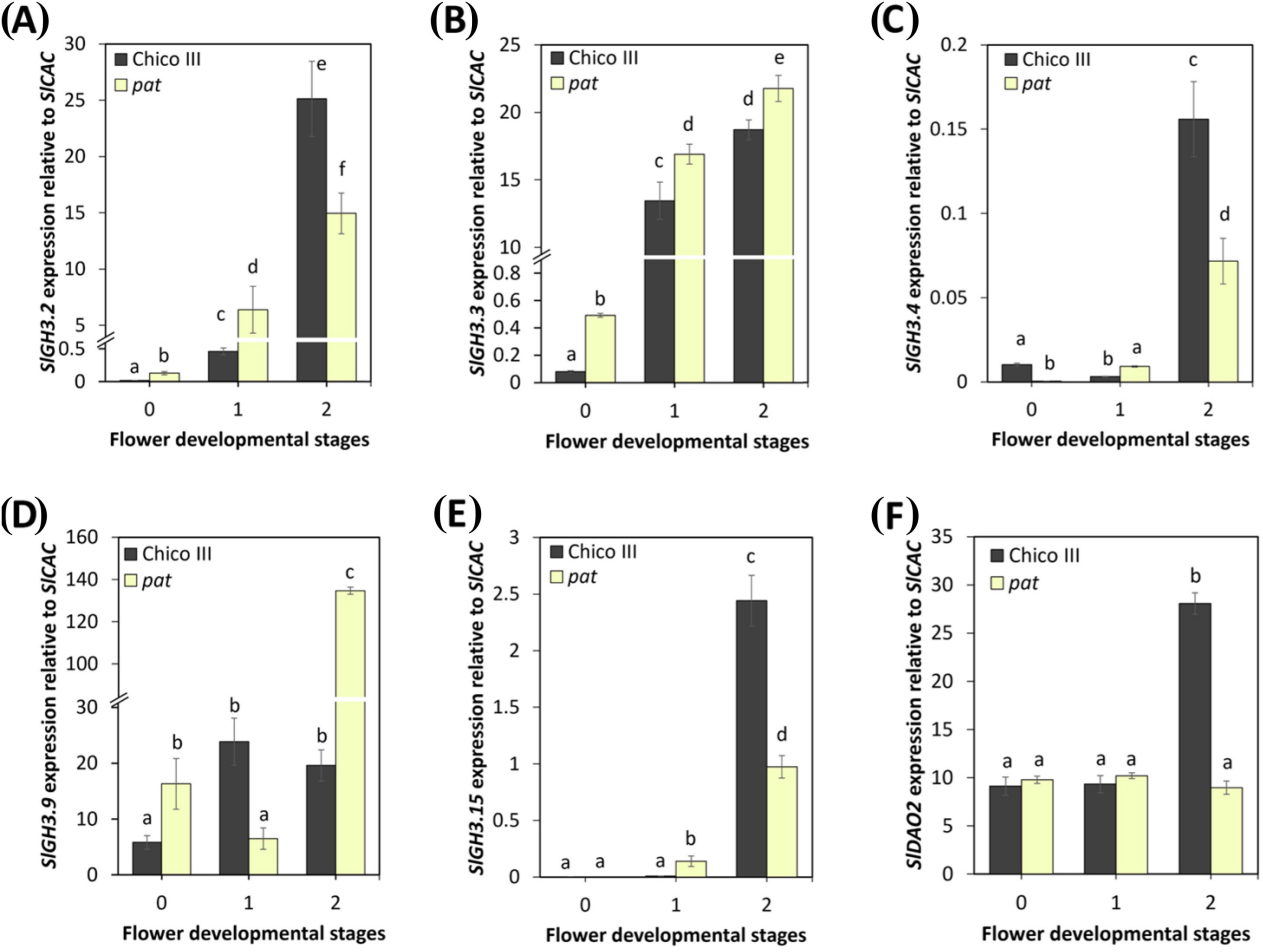
*SlGH3s* and *SlDAO2* expression in *pat* and wild type (Chico III) stamens. qRT‐PCR analysis of *SlGH3.2* (A), *SlGH3.3* (B), *SlGH3.4* (C), *SlGH3.9* (D), *SlGH3.15* (E) and *SlDAO2* (F) expression in *pat* and Chico III stamens at stages from 0 to 2 of flower development. Genes expression is presented relative to the reference gene *SlCAC*. The values shown are means ± SD (*n* = 3). Bar colors indicate different lines (i.e., Chico III, black; *pat*, light yellow). Different letters represent significant differences as determined by two‐way ANOVA followed by Tukey's test (*p* < 0.05).

According to the previous section, we speculated that *SlGH3s* and *SlDAO2* genes could be involved in the regulation of stamen growth in tomato, similarly to Arabidopsis. To verify this hypothesis, we compared the expression of group II *SlGH3s* genes in Chico III and *pat* stamens at stages 0, 1 and 2 of flower development, corresponding to stages 9–11 in Arabidopsis. As shown in Figure 7, the expression of *SlGH3.2* and *SlGH3.3* was significantly increased at stages 0 and 1 in *pat* mutant stamens compared to Chico III, while it increased and decreased significantly, respectively, at stage 2 (Figure 7A,B). The expression of *SlGH3.4* in *pat* stamens was decreased at stage 0, slightly increased at stage 1 and decreased again at stage 2, compared to Chico III (Figure 7C). The expression of *SlGH3.9* in *pat* stamens was upregulated at stages 0 and 2, and downregulated at stage 1 (Figure 7D), while that of *SlGH3.15* was upregulated at stage 1 and repressed at stage 2 compared to Chico III (Figure 7E). In addition, we analyzed the expression of *SlDAO2* in *pat* mutant stamens at stages 0–2 of flower development and we found that it remained unchanged at stages 0 and 1, while it significantly decreased at stage 2, compared to Chico III (Figure 7F).

These results indicate that all *SlGH3s* genes are deregulated in *pat* stamens, most of them being upregulated at stages 0 and 1, while *SlDAO2* is downregulated only at stage 2, suggesting that the conjugation activity of SlGH3, on IAA or JA, might be increased, while DAO2‐dependent IAA oxidizing activity is decreased in *pat* stamens.

### 3.8 Tomato *pat* Mutants Have a Decreased Auxin Content in Flower Buds

**FIGURE 8:**
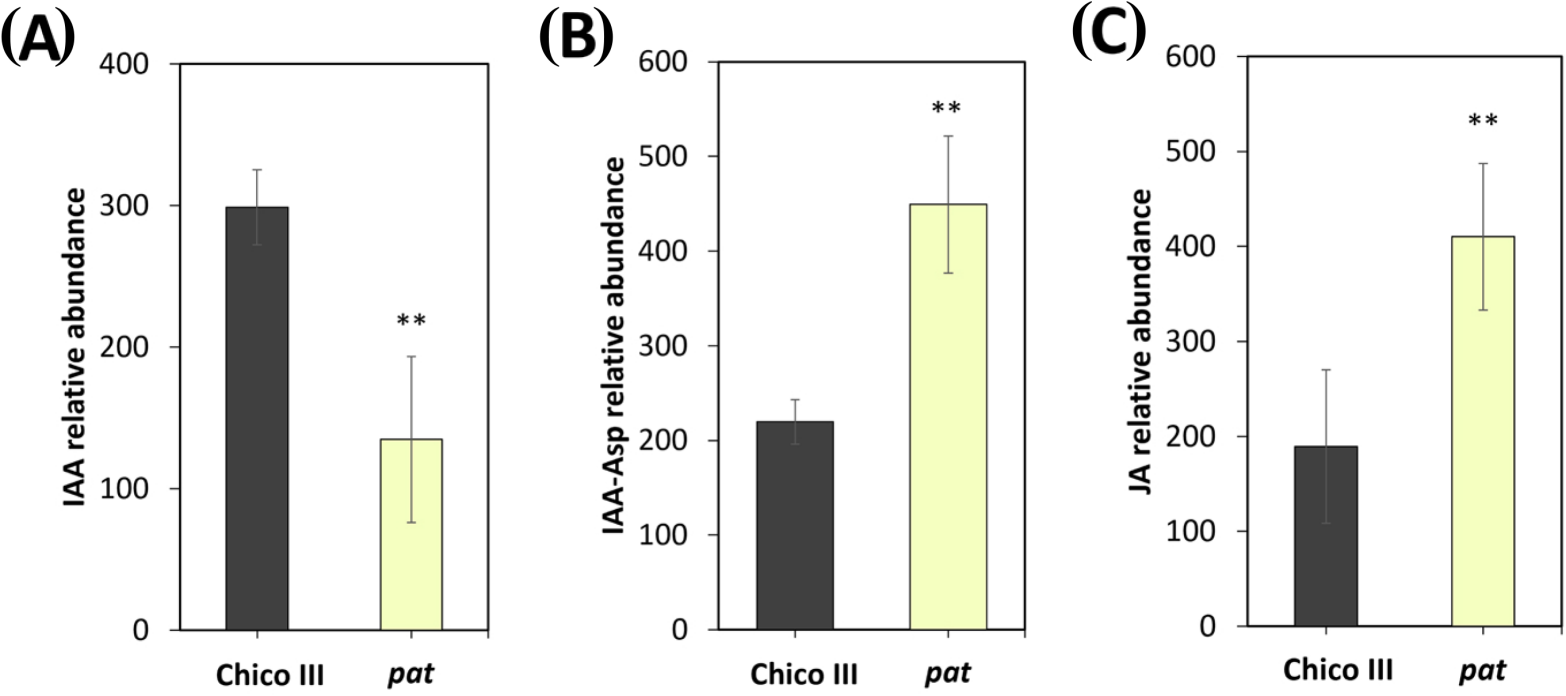
Analysis of IAA, IAA‐Asp and JA concentration in *pat* and wild type (Chico III) stamens. Hormones and hormones conjugate analysis was performed on 20 mg of stamens at stages 1–2 pooled together of flower development, collected from *pat* mutant and Chico III lines. Relative amount concentration of IAA (A), IAA‐Asp (B) and JA (C) are reported. Values are the means of three biological replicates ± SD. Bar colors indicate different lines (i.e., Chico III, black; *pat*, light yellow). Statistically significant differences were determined by Student's *T* test and indicated by asterisks (**p < 0.01).

To evaluate whether *SlGH3* and *SlDAO1* gene deregulation affects hormone content in the *pat* mutants, we compared the amount of free endogenous IAA, IAA‐Asp, and JA levels in Chico III and *pat* stamens at stages 1–2 (pooled together), as auxin and JA are highly detectable in pre‐anthesis stages in tomato (Gillaspy et al. 1993; Khaliluev et al. 2014). As shown in Figure 8, IAA levels significantly decreased in *pat* stamens compared to Chico III ones (Figure 8A), while IAA‐Asp abundance was significantly increased (Figure 8B). Interestingly, in contrast to Arabidopsis, JA content was significantly increased in *pat* mutant stamens compared to the control (Figure 8C). These results indicate that in tomato, similarly to Arabidopsis, *SlGH3s* and *SlDAO1* play a central role during stamen maturation, regulating not only auxin homeostasis but also JA levels to achieve appropriate organ development.

## 4 Discussion

Late stamen development is regulated by auxin through changes in auxin concentration and localization, during subsequent developmental stages (Cardarelli and Cecchetti 2014). The biosynthesis and distribution of auxin in different stamen tissues have been extensively studied (Cardarelli and Cecchetti 2014; Cecchetti et al. 2013, 2008). However, the role of the main auxin inactivation pathways, which include conjugation to amino acids by GH3s and oxidation by DAO1, remains elusive.

In this study, we show through qRT‐PCR analysis that *AtGH3.3*, *AtGH3.5*, and *AtGH3.6* are downregulated, whereas *AtDAO1* is upregulated in *arf8‐7* stamens during late flower development (Figures 1, 2, 3), indicating the involvement of these genes in ARF8‐controlled stamen filament elongation. These data are consistent with previous reports on the role of ARF8 in auxin signaling in reproductive organs (Ghelli et al. 2018; Nagpal et al. 2005). By analyzing the expression of *AtGH3.3*, *AtGH3.5*, and *AtGH3.6* in Col‐0 stamens, the phenotype of the *gh3.3 gh3.5 gh3.6* triple mutants flowers, as well as free and conjugated IAA levels in flower buds, we demonstrated that *GH3s* modulate free auxin levels mainly during early stages of late flower development (stages 10 and 11) by catalyzing its conjugation to amino acids. Indeed, the auxin‐inducible genes *AtGH3s* are expressed in Col‐0 stamens with partially overlapping expression profiles, and their transcript levels are detected mainly at stage 10 when auxin concentration is high, and at stage 11 when it starts to decrease (Hayashi et al. 2021). Among *GH3s*, *AtGH3.3* and *AtGH3.5* were the most expressed during stamen development, while *AtGH3.6* was expressed at lower levels (Figure 1). Consistently, the significant increase in free IAA and the concomitant decrease in IAA‐Asp in *gh3.3 gh3.5 gh3.6* triple mutant flower buds provide direct evidence that AtGH3s catalyze auxin conjugation during late development (Figure 4). In agreement with the observed increase in free IAA and the promotive role of auxin on filament elongation, the *gh3.3 gh3.5 gh3.6* triple mutant flowers exhibit stamens that are 24.8% longer than Col‐0 stamens at stage 14. Our results confirm previous studies that demonstrated the role of AtGH3s in catalyzing IAA conjugation to amino acids and in particular in IAA‐Asp formation (Ludwig‐Müller 2011; Staswick et al. 2005). Furthermore, by measuring the content of free JA, which is found to be unchanged in *gh3.3 gh3.5 gh3* triple mutants versus Col‐0 flower buds, we ruled out the possibility that the *AtGH3s* genes act on the formation of JA conjugates during early stages of stamen late development, as observed for adventitious root formation (Gutierrez et al. 2012).

We also found that auxin levels in stamens require a fine‐tuned balance between both conjugation and degradation via *AtGH3s* and via *AtDAO1*, respectively. This is achieved by the compensatory gene expression induced by mutations in individual *AtGH3s* genes that activate or inhibit the transcription of the other *AtGH3*s, as well as that of *AtDAO1*. Indeed, altered expression of other *AtGH3s* and *AtDAO1* is observed in all single mutants that have shorter stamens than Col‐0. On the other hand, the upregulation of *AtGH3.3*, *AtGH3.5*, and *AtGH3.6* in *dao1* mutants (Figure 3) suggests a feedback loop in which decreased auxin degradation triggers increased conjugation by GH3s, possibly as a compensatory response to restore auxin homeostasis. However, the absence of all three *AtGH3s* in the *gh3s* triple mutant promotes stamen elongation and triggers the downregulation of *AtDAO1*, consistent with AtDAO1 acting downstream of AtGH3s by oxidizing the IAA‐Asp and IAA‐Glu conjugates produced by AtGH3s (Hayashi et al. 2021). Consistently, *AtDAO1* expression in stamens was mainly observed at stage 11, whereas *AtGH3s* are mainly expressed at stage 10, corresponding to auxin concentration maxima in stamens (Cecchetti et al. 2013, 2008). Furthermore, since auxin negatively regulates JA biosynthesis by acting on both JA biosynthetic genes, *AtDAD1* and *AtOPR3*, JA levels at stage 11 increase in Arabidopsis wild type stamens (Cecchetti et al. 2015). This suggests that JA may induce *AtDAO1* expression in stamens, as previously shown during adventitious root formation (Lakehal, Dob, et al. 2019).

We also show that *dao1‐1* and *dao1‐3* stamens are 21% and 16.2% shorter than Col‐0 stamens, respectively, likely due to the upregulation of *AtGH3s* in *dao1* mutants (Figure 3), as GH3s have been shown to respond rapidly to environmental factors that increase cellular IAA levels (Zhang et al. 2016).

To assess whether the *ARF8*‐*GH3s*‐*DAO1* module is conserved during tomato stamen development, we identified five *SlGH3s* members belonging to group II as well as *SlDAO2*, which is homologous to *AtDAO1*. We found that SlGH3.2, SlGH3.3, and SlGH3.4 have the highest percentage of protein identity to AtGH3.3, and SlGH3.9 and SlGH3.15 the highest percentage of protein identity with AtGH3.5 and AtGH3.6 (Figure S2; Table S3). Similar to Arabidopsis, by analyzing the expression of five *SlGH3* members in Chico III tomato stamens and in the *pat* mutant, which is defective in stamen growth, free and conjugated auxin, and JA content in stamens, we show that the *SlGH3* genes are involved in auxin conjugation during stamen development also in tomato. Indeed, all these genes are expressed in Chico III stamens with partially overlapping profiles, as *SlGH3.2, SlGH3.3*, and *SlGH3.9* transcript levels are mainly high at stages 1 and 2, roughly corresponding to Arabidopsis stages 10 and 11, while *SlGH3.15* and *SlGH3.4* are mainly expressed later at stages 3 and 4 (Figure 5). In agreement with the reduced stamen elongation phenotype, *pat* mutant stamens show reduced free IAA, increased IAA‐Asp, and an increased expression at stage 0 of *SlGH3.2, SlGH3.3*, and *SlGH3.9*, while *SlGH3.4* and *SlGH3.15* are upregulated at stage 1. Our data also suggest a role for ARF8 in proper stamen development in tomato. Indeed, the *AtARF8* orthologs *SlARF8A* and *SlARF8B* are expressed in Chico III stamens at stages 0 and 1, whereas they are downregulated in *pat* stamens (Figure 6), consistent with the impaired auxin homeostasis and reduced stamen elongation observed in these mutants (Liu et al. 2014; Zouine et al. 2014). The loss of SlARF8 in tomato is consistent with the role of AtARF8 in Arabidopsis, where it is known to regulate stamen filament elongation (Ghelli et al. 2018; Nagpal et al. 2005; Tabata et al. 2010). These data are also consistent with recent findings showing that *pat* is characterized by a mutation in the HD‐ZIP gene *SlHB15A*, which has a pleiotropic phenotype and affects auxin signaling, deregulating the expression of several auxin‐related genes such as *SlARF8*, *SlARF9*, *SlIAA9*, and *SlIAA14* (Clepet et al. 2021; Picarella et al. 2024; Ruiu et al. 2015). A direct interaction between *pat* and *SlARF7* has been demonstrated (Clepet et al. 2021), and the downregulation of *SlARF8* at early stages in the *pat* ovary has been also shown (Ruiu et al. 2015). Therefore, it is likely that *pat* acts upstream of both *SlARF* transcription factors to control auxin‐inactivating enzymes.

However, in contrast to Arabidopsis, the reduced stamen length and other developmental defects observed in the *pat* mutants are likely a consequence of disrupted auxin as well as of JA homeostasis. Furthermore, auxin and JA have antagonistic effects during the abscission process that occurs in tomato flowers (Liu, Cheng, et al. 2022). The increase in JA content correlates with the misregulation of *SlGH3* and *SlDAO2* in *pat* tomato stamens. This is consistent with the different role of JA in stamen development in Arabidopsis and tomato, which is probably due to the primary role of this hormone in ovary development in tomato. Indeed, the Arabidopsis *coi1* mutant, defective in JA perception, shows reduced stamen elongation and anther dehiscence, as well as non‐functional pollen, whereas the tomato jasmonic‐acid insensitive1 (*jai1*) mutant which is defective in the ortholog of Arabidopsis *COI1*, shows reduced pollen viability and female sterility, without any defect in anther dehiscence (Niwa et al. 2018). However, in tomato flower buds, auxin accumulation starts from stages 0 and 1 (Goldental‐Cohen et al. 2017; Mazzucato et al. 1998), while JA is detectable from stage 2 (Niwa et al. 2018), suggesting that downregulation of *SlGH3.4*, which occurs at stages 1 and 2 in *pat* mutant stamens reduces JA conjugation, thus leading to an increase in free JA. Consistent with this, the role of AtGH3s in JA conjugation has already been demonstrated in Arabidopsis during adventitious root formation (Gutierrez et al. 2012). However, we cannot rule out the possibility that the elevated JA levels in tomato stamens are due to altered expression of *SlGH3s* other than those homologous to *AtGH3s*. Further work will be necessary to unveil the specific role of JA in relation to GH3s in tomato stamen development.

## 5 Conclusions

This study provides insights into the role of *AtGH3s* and *AtDAO1* genes in stamen development, highlighting the compensatory regulation among these genes and their involvement in the regulation of auxin homeostasis. Our results reveal that *AtGH3s* regulate stamen elongation by conjugating auxin and thus reducing free IAA at the initial stages of late flower development, immediately after the auxin maximum. In contrast, *AtDAO1* acts predominantly at the final stages of flower development. We also show that *AtGH3s* and *AtDAO1* orthologs regulate auxin homeostasis in tomato stamens, highlighting the evolutionary conservation of these processes. The stable JA levels observed in Arabidopsis *gh3s* mutants stamens exclude a potential role for *AtGH3s* in JA conjugation in this plant species. In contrast, the elevated JA levels in the tomato *pat* mutant suggest that *SlGH3s* are involved in the regulation of JA homeostasis during tomato stamen development. These findings are consistent with the different roles of JA in male and female fertility across Arabidopsis and tomato species.

## Author Contributions

M.C. and P.B. conceived and designed the research. D.M. and P.B. performed the research. D.M., M.L.A., R.G., V.C., F.R.I., M.B., M.E.P., and P.B. conducted the experiments. D.M. and P.B. contributed to the data analysis. M.C., D.M., and P.B. performed the data analysis interpretation. D.M., P.B., and M.C. wrote the manuscript. D.M., P.B., M.C., A.M., and M.R. revised the manuscript. All authors read and approved the manuscript.

## Conflicts of Interest

The authors declare no conflicts of interest.

## Supporting information


**Figure S1.** *AtGH3s* expression in Arabidopsis wild type (Col‐0) and *arf8‐7* stamens.
**Figure S2.** Phylogenetic analysis of three AtGH3.3, AtGH3.5, AtGH3.6 genes and the other tomato homologs from the GH3 multigene family.
**Table S1.** Gene specific primers used for this study.
**Table S2.** NCBI Accession Number of protein sequences used for phylogenetic analysis.
**Table S3.** Amino acid sequence identity of *Solanum Lycopersicum* and *Arabidopsis thaliana* proteins addressed in this study.
**Table S4.** MRM conditions used in this study.
**Table S5.** Correspondence of the Arabidopsis and tomato floral stages considered in the manuscript and reproductive landmark events.

## Data Availability

The data that supports the findings of this study are already contained within this article.
