# Supplementary material for: Primary Auxin Response Genes GH3s and DAO1 Modulate Stamen Elongation in Arabidopsis thaliana and Solanum lycopersicum
Source: Physiol Plant. 2025 Jun 18;177(3):e70340. doi: 10.1111/ppl.70340 (PMC12177280; doi:10.1111/ppl.70340)
Supplement: Supplementary file 1 — Figure S1. AtGH3s expression in Arabidopsis wild type (Col‐0) and arf8‐7 stamens. Figure S2. Phylogenetic analysis of three AtGH3.3, AtGH3.5, AtGH3.6 genes and the other tomato homologs from the GH3 multigene family. Table S1. Gene specific primers used for this study. Table S2. NCBI Accession Number of protein sequences used for phylogenetic analysis. Table S3. Amino acid sequence identity of Solanum Lycopersicum and Arabidopsis thaliana proteins addressed in this study. Table S4. MRM conditions used in this study. Table S5. Correspondence of the Arabidopsis and tomato floral stages considered in the manuscript and reproductive landmark events. [file PPL-177-e70340-s001.docx]

Supporting Information

Primary auxin response genes *ARF8, GH3s* and *DAO1* modulate stamen elongation in *Arabidopsis thaliana* and *Solanum lycopersicum*

Davide Marzi^1,2^, Maria Luisa Antenozio^1^, Roberta Ghelli^3^, Valentina Cecchetti^4^, Francesca Romana Iacobini^5^, Marzia Beccaccioli^4^, Massimo Reverberi^4^, Maurizio Enea Picarella^6^, Andrea Mazzucato^6^, Patrizia Brunetti^1,*^ and Maura Cardarelli^3^

**1. Supporting materials and methods**

**Phylogenetic Analysis**

Multiple sequence alignment was performed by using ATGH3.3, AtGH3.5, AtGH3.6 protein sequence (TAIR <https://www.arabidopsis.org/>) and the only *Solanum Lycopersicum* thirteen homolog proteins sequence available from both NCBI (<https://www.ncbi.nlm.nih.gov>) and Sol Genomic Network (<https://solgenomics.net>) with MUSCLE in MEGA11 phylogeny program (Stecher et al., 2020; Tamura et al., 2021) with default gap penalties. An un-rooted neighbor- joining phylogenetic tree was constructed using the Neighbor–Joining method within the MEGA 11. Bootstrap analysis was carried out taking 1,000 replicates (Fig. S2).

**2. Tables**

**Table S1.** Gene specific primers used for this study.

| **Primers used for PCR** | | |
| --- | --- | --- |
| *gh3.3-1 For* | TTTTAACGTATTAATCTTGGCACG | **Gutierrez et al., 2012** |
| *gh3.3-1 Rev* | GGGAACAACAACATGATCCCT |  |
| *gh3.5-2 For* | CACACCTTGTCCCATTTGATG |  |
| *gh3.5-2 Rev* | TGTGGCTTAATTGTATGTGTGTCA |  |
| *gh3.6-1 For* | GCTTAGAGAAACATAAACCGGCTA |  |
| *gh3.6-1 Rev* | GACTTCTTGGCAAGGGATCA |  |
| *dao1-1 For* | TTCCCCACGGAATTAAGGTAC | **Zhang et al., 2016** |
| *dao1-1 Rev* | CAAGTCCATTGATAGCCTTCG |  |
| *dao1-3 For* | GGTCTGAATTGGACAATGACG |  |
| *dao1-3 Rev* | CTTGAGAAGTAGCATCAGTTTCTG |  |
| **Primer used for Real-time qRT-PCR** | | |
| *AtGH3.3 For* | ACAATTCCGCTCCACAGTTC | **Gutierrez et al., 2012** |
| *AtGH3.3 Rev* | ACGAGTTCCTTGCTCTCCAA |  |
| *AtGH3.5 For* | GTCTTCGAGGACTGCTGCTT |  |
| *AtGH3.5 Rev* | ATGTCCCTGGCTCAACAATC |  |
| *AtGH3.6 For* | CCTTGTTCCGTTTGATGCTT |  |
| *AtGH3.6 Rev* | CGTGTTACCGTTCAAGCAGA |  |
| *AtDAO1 For* | GTTACAGAGCTCCAAACGAAA | **This study** |
| *AtDAO1 Rev* | TCCGTTGCAAGTCCATTGA |  |
| *SlGH3.2* For | GTGAACTTTGCACCTATT | **Liao et al., 2015** |
| *SlGH3.2* Rev | AAACACTTCTCCTCCTCT |  |
| *SlGH3.3* For | TACACCTATAGCGGAATT |  |
| *SlGH3.3* Rev | TATGTGGTCTCGATCATG |  |
| *SlGH3.4* For | CTCCAGGGTGATTTCTGT |  |
| *SlGH3.4* Rev | TTCTTTGGTCCACTGTCT |  |
| *SlGH3.9* For | ACAAGCAATGGAACAACA |  |
| *SlGH3.9* Rev | GGGTCAACCTAACCAAGA |  |
| *SlGH3.15* For | GCACCCATTATTGAACTA |  |
| *SlGH3.15* Rev | TCTTGGACTTATGATGAAGC |  |
| *SlARF8A For* | TGACATCGAATGGAAATTCAG | **Zouine et al., 2014** |
| *SlARF8A Rev* | GTCTCTTAGCACTAACAAACAC |  |
| *SlARF8B For* | GTCAGTCCGTGATCATAGAG |  |
| *SlARF8B Rev* | GGAATCCAAGCTACAATTTCC |  |
| *SlDAO2 For* | TGGGACCAAGGGACACGGCA | **This study** |
| *SlDAO2 Rev* | AGAGCTTCCCCTGCCTGCAACT |  |
| *SlCAC For* | GATGTCCTTATCAACCGTCTCTAC | **Ruiu et al., 2015** |
| *SlCAC Rev* | ACAAGAAAGAACAGCCTCCAATCT |  |

**Table S2.** NCBI Accession Number of protein sequences used for phylogenetic analysis. The Relative SolGenomics gene accession number is indicated.

| **NCBI Protein name** | **NCBI Accession Number** | **SolGenomics accession Number** |
| --- | --- | --- |
| SlGH3.1 | XP_004231204.3. | Solyc01g095580.3.1 |
| SlGH3.2 | NP_001308616.1 | Solyc01g107390.2 |
| SlGH3.3 | XP_004233446.1 | Solyc02g064830.2 |
| SlGH3.4 | XP_004231891.1 | Solyc02g092820.4.1 |
| SlGH3.5 (JAR4) | XP_004240084.2 | Solyc05g050280.3.1 |
| SlGH3.7 | XP_010327157.1 | Solyc10g006610.4.1 |
| SlGH3.8 | XP_004243319.2 | Solyc07g054580.3.1. |
| SlGH3.9 | XP_004244168.1 | Solyc07g063850.2 |
| SlGH3.10 | NP_001355237.1 | Solyc10g008520.3.1 |
| SlGH3.12 | XP_004248077.1. | Solyc10g009610.2.1 |
| SlGH3.13 (Jar6) | XP_004248076.1 | Solyc10g011660.3.1 |
| SlGH3.14  (Jar1) | XP_004248075.1 | Solyc10g011660.3.1 |
| SlGH3.15 | XP_004251485.1. | Solyc12g005310.2.1 |
| SlGH3.17 | XP_004243428.2 | Not available |
| SlGH3.18 | Not available | Not available |
| SlGH3.20 | Not available | Not available |

**Table S3.** Amino acid sequence identity of *Solanum Lycopersicum* and *Arabidopsis thaliana* proteins addressed in this study.

| **Arabidopsis gene name and locus (TAIR)** | **Tomato gene name and locus**  **(SolGenomics, Liao et al., 2015)** | **Identity (%)**  **(SolGenomics, JGI Phytozome)** | **Identity alignment**  **(Arabidopsis/tomato exact amino acid matches)** | **Score** |
| --- | --- | --- | --- | --- |
| *AtGH3.3*  AT2G23170.1 | *SlGH3.2* Solyc01g107390.2 | 76.63 | 459/599 | 945 |
|  | *SlGH3.3*  *Solyc02g064830.2* | 73.91 | 442/598 | 924 |
|  | *SlGH3.4*  Solyc02g092820.2 | 73.38 | 441/601 | 922 |
| *AtGH3.5*  AT4G27260.1 | *SlGH3.9*  Solyc07g063850.2 | 81.7 | 500/612 | 1046 |
|  | *SlGH3.15*  Solyc12g005310.1 | 79.12 | 485/512 | 1016 |
| *AtGH3.6* AT5G54510.1 | *SlGH3.9*  Solyc07g063850.2 | 83.01 | 508/612 | 1072 |
|  | *SlGH3.15*  Solyc12g005310.1 | 80.42 | 493/613 | 1031 |
| *AtDAO-1*  AT1G14130.1 | *SlDAO-2*  *Solyc02g068320.2* | 58.98 | 174/295 | 352 |
| *AtDAO-2*  *AT1G14120* | *SlDAO-2*  *Solyc02g068320.2* | 52.48 | 159/303 | 314 |
| *AtARF8*  AT5G37020.1 | *SlARF8A*  *Solyc03g031970.2* | 67.31 | 558/829 | 1046 |
|  | *SlARF8B*  *Solyc02g037530.2* | 67.84 | 557/821 | 1056 |

**Table S4**. MRM conditions used in this study.

| Analyte | Transition | Fragmentor (V) | Collision energy (eV) | Polarity |
| --- | --- | --- | --- | --- |
| JA | 209.2→59.1 | 135 | 28 | [M-H]^-^ |
| IAA | 174→130.1 | 70 | 7 | [M-H]^-^ |
| IAA-Asp | 291.1→130.1 | 46 | 25 | [M+H]^+^ |
| NAA | 185→141 | 100 | 12 | [M-H]^-^ |

**Table S5.** Correspondence of the Arabidopsis and tomato floral stages considered in the manuscript and reproductive landmark events.

| **Arabidopsis flower development (Smyth et al., 1990)** | | **Tomato development (Mazzucato et al., 1998)** | | **Arabidopsis stamen event (Cardarelli and Cecchetti, 2014)** |
| --- | --- | --- | --- | --- |
| **Floral stage** | **Landmark event / phenotype** | **Floral stage** | **Landmark event / phenotype^a^** |  |
| 9 | Petal primordia stalked at base | 0 | Flower bud 3.0 to 5.9 mm long | Microsporogenesis |
| 10 | Petals level with short stamens | 1 (early) | Flower bud 6.0 to 8.9 mm long | Callose degeneration, microspore release, filament elongation starts |
| 11 | Stigmatic papillae appear | 1 (late) | Flower bud 6.0 to 8.9 mm long | Tapetum and middle layer degeneration, endothecium lignification, mitosis I |
| 12 | Petals level with long stamens | 2 | Flower bud 9.0 to 12.0 mm long | Filament elongation, septum lysis  mitosis II |
| 13 | Bud opens, petals visible, anthesis | 3 | Opening flower | Anthesis starts, breakage of the stomium |
| 14 | Long anthers extend above stigma | 4 | Anthesis | Anthesis, pollination |

^a^ Bud size refers to flowers of the cv. Chico III genetic background

**3. Figures**

**Fig. S1. *AtGH3s* expression in Arabidopsis wild type (Col-0) and *arf8-7* stamens.** qRT-PCR analysis of *AtGH3.3* (A), *AtGH3.5* (B) and *AtGH3.6* (C) expression in wild type (Col-0) and *arf8-7* mutant stamens at stages 10-12 (pooled together) of flower development. Genes expression is normalized to Col-0 and indicated as fold change. The values shown are means ± SD (n=3). Bar colors indicate different lines (i.e., Col-0, black; arf8-7, white). Statistically significant differences were determined by Student’s T test and indicated by asterisks (** *P*<0.01, *** *P*<0.001).


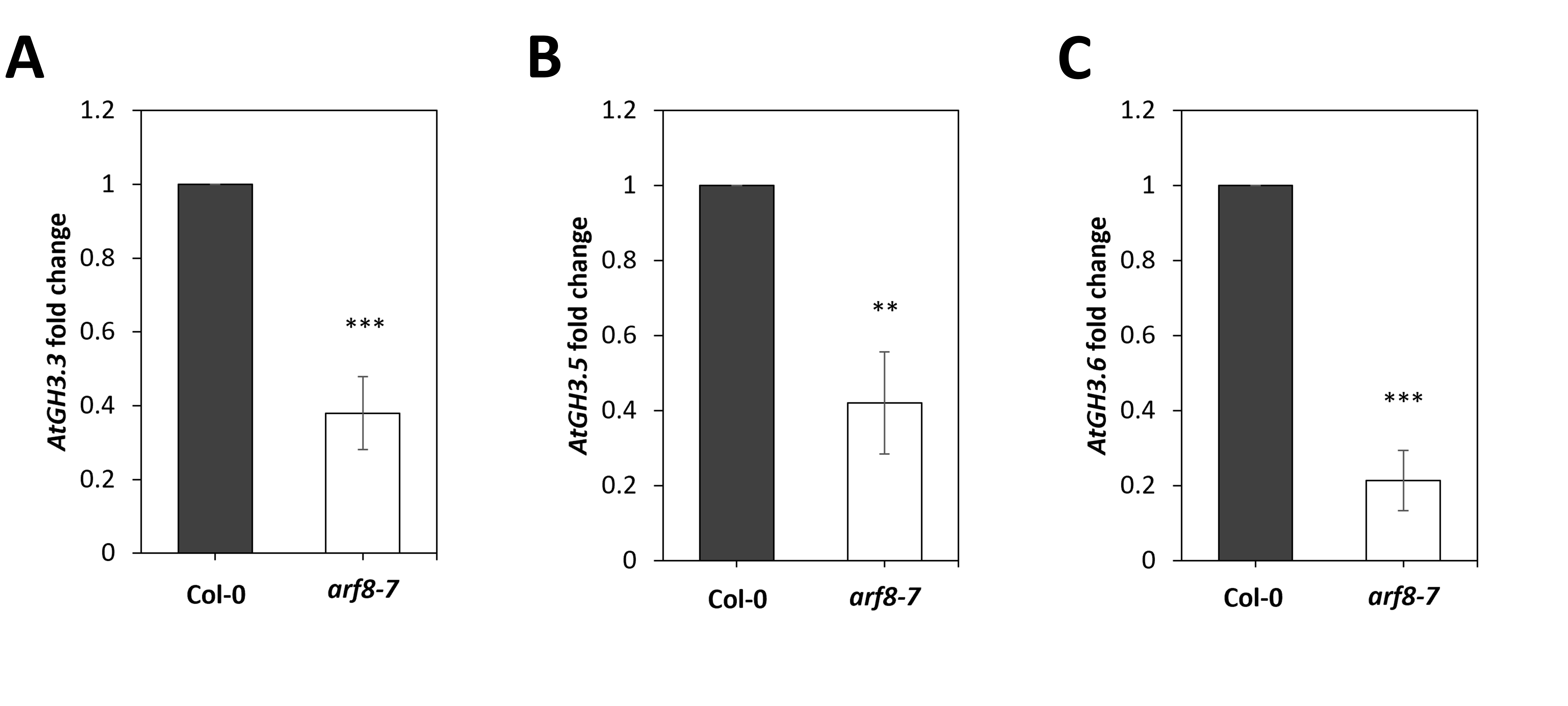

**Fig. S2.** Phylogenetic analysis of three AtGH3.3, AtGH3.5, AtGH3.6 genes and the other tomato homologs from the GH3 multigene family. The accession numbers for the related GH3 genes are listed in Tabe S2. Ellipses with the same color indicate the two subgroups, belonging to the GROUP II Family, with high bootstrap values (black for AtGH3.3, SlGH3.2 SlGH3.3, SlGH3.4 and red/blue for AtGH3.5, AtGH3.6, SlGH3.9, SlGH3.15 and SlGH3.7).

**References**

Stecher, G., Tamura, K., & Kumar, S. (2020). Molecular evolutionary genetics analysis (MEGA) for macOS. *Molecular Biology and Evolution*, *37*(4), 1237–1239. https://doi.org/10.1093/molbev/msz312

Tamura, K., Stecher, G., & Kumar, S. (2021). MEGA11: Molecular Evolutionary Genetics Analysis Version 11. *Molecular Biology and Evolution*, *38*(7), 3022–3027. https://doi.org/10.1093/molbev/msab120
